# The Enigmatic HOX Genes: Can We Crack Their Code?

**DOI:** 10.3390/cancers11030323

**Published:** 2019-03-07

**Authors:** Zhifei Luo, Suhn K. Rhie, Peggy J. Farnham

**Affiliations:** Department of Biochemistry and Molecular Medicine and the Norris Comprehensive Cancer Center, Keck School of Medicine, University of Southern California, Los Angeles, CA 90089, USA; zhifeilu@usc.edu (Z.L.); rhie@usc.edu (S.K.R.)

**Keywords:** HOX, ChIP-seq, cancer biomarkers, targeted therapy

## Abstract

Homeobox genes (HOX) are a large family of transcription factors that direct the formation of many body structures during early embryonic development. There are 39 genes in the subgroup of homeobox genes that constitute the human HOX gene family. Correct embryonic development of flies and vertebrates is, in part, mediated by the unique and highly regulated expression pattern of the HOX genes. Disruptions in these fine-tuned regulatory mechanisms can lead to developmental problems and to human diseases such as cancer. Unfortunately, the molecular mechanisms of action of the HOX family of transcription factors are severely under-studied, likely due to idiosyncratic details of their structure, expression, and function. We suggest that a concerted and collaborative effort to identify interacting protein partners, produce genome-wide binding profiles, and develop HOX network inhibitors in a variety of human cell types will lead to a deeper understanding of human development and disease. Within, we review the technological challenges and possible approaches needed to achieve this goal.

## 1. Introduction

The homeobox gene family is the second largest family of transcription factors encoded in the human genome and consists of an estimated 257 genes [1], each of which contains a 183-nucleotide sequence that encodes a 61-amino acid homeodomain that forms a helix-turn-helix structure. The subgroup of homeobox genes that constitute the human HOX gene family consists of 39 homeobox genes. Initial insights into the role of HOX genes in development came from studies of Drosophila HOX proteins, which include nine genes divided into two sub-clusters [2]. However, more recent studies have shown that HOX genes play an important role in determining cell identity and body formation along the anterior and posterior axes in many types of animals; although homeobox genes are encoded in plant genomes, the HOX subgroup is specific to animals [3,4,5,6]. 

**Figure 1:**
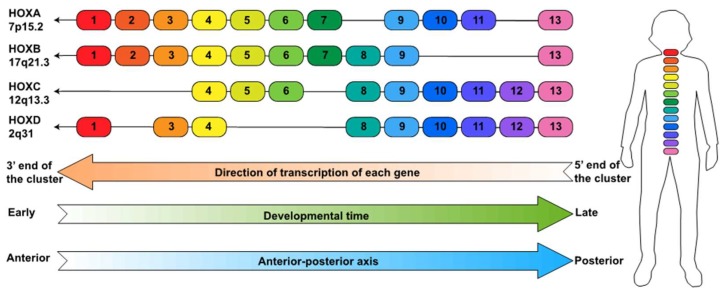
The HOX loci. Shown are the HOX family members present in each of the four loci, along with the direction of individual gene transcription and the directions of the spatial and temporal waves of transcription of the genes in each cluster; shown on the right is a schematic indicating the relative positions in the human body at which the HOX paralogs are expressed during development.

The human HOX genes are located at four chromosomal loci; HOXA is at 7p15.2, HOXB is at 17q21.3, HOXC is at 12q13.3 and HOXD is at 2q31 (Figure 1). The HOX genes are numbered from 1 to 13 in each locus. However, each locus has lost several genes during evolution such that there are only three numbered HOX paralogs (HOX4, HOX9, and HOX13) that are present at all four HOX loci. Mutations in 10 of the 39 HOX genes (HOXA1, HOXA2, HOXA11, HOXA13, HOXB1, HOXB13, HOXC13, HOXD4, HOXD10, and HOXD13) have been found to cause human disorders with significant variation in their inheritance patterns, penetrance, and pathogenesis (reviewed in [7]). For example, heterozygous mutations in HOXA13 cause hand–foot–genital syndrome (HFGS) and Guttmacher syndrome. HFGS is an autosomal dominant condition characterized by limb malformations and urogenital defects, whereas Guttmacher syndrome has similar limb malformations and urogenital defects as HFGS but also includes additional hand defects. Similarly, HOXD13 mutations result in a variety of malformations in the fingers or toes.

**Figure 2:**
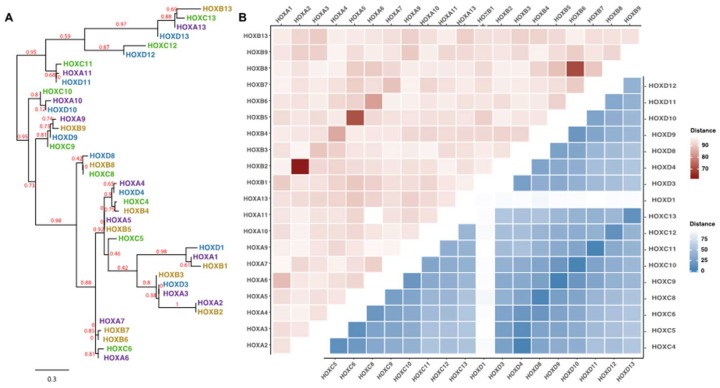
Phylogenetic analysis of HOX genes. (**A**) Shown is a phylogram using the full-length protein sequences of the HOX family members generated by phlogeny.pr [9,10]. The bootstrap value, representing the reproducibility of the tree structure, is shown for the horizontal branches; the scale bar indicates the length representing 0.3 substitutions per site. All HOX family members from a given locus are in the same color. (**B**) Shown is a distance matrix comparing the full-length protein sequences from the HOXA locus to the full-length protein sequences from the HOXB locus (left top triangle; pink squares) and a distance matrix comparing the homeodomain sequences of the HOXC proteins versus the homeodomain sequences of the HOXD proteins (right bottom triangle; blue squares); matrices were created using distmat [11]. Color legend: Distance: the darker the color, the more similar are the two proteins and the lighter the color the less similar are the two proteins. The HOX sequences were retrieved from RefSeq [12] and the homeodomains were annotated using Pfam [13]. A full distance matrix comparing all 39 HOX proteins (full-length and homeodomains) is shown in Figure S1; see Table S1 for all distance values.

Phylogenetic analyses indicate that the same numbered genes in each of the four HOX loci (i.e., the paralogs) are more similar to each other than they are to adjacent genes in the same loci. For example, Figure 2A shows that HOXB8 is clustered with HOXC8 and HOXD8, but quite far from HOXB7 and HOXB9 (the two adjacent genes at the same loci). Similarly, a distance matrix analysis reveals that HOXA2 is very closely related to HOXB2 and HOXA5 is very closely related to HOXB5 (Figure 2B, top, pink section). This relationship between paralogs is even more striking when the homeodomains are compared (Figure 2B, bottom, blue section). The high degree of similarity amongst the HOX genes suggests that the DNA binding domains (and consequently the target binding sites) are under strong evolutionary selection pressure. The similarity between paralogs also raises the interesting possibility that perhaps they can substitute for each other under certain situations (e.g., functional loss of one paralog could be compensated for by increased expression of another paralog). In fact, there is evidence for functional redundancy in kidney development between the members of the HOX11 paralogs. Mice missing one *Hox11* gene have normal kidneys, mice missing any two *Hox11* genes have hypoplasia of the kidney, and knockout of the entire *Hox11* paralogous gene group abolishes the initiation of kidney development [8].

Clearly, HOX proteins regulate cell adhesion, division, death, migration and shape in their roles in defining morphology and therefore they must control genes involved in these pathways, probably by binding to regulatory elements that control activity of the promoter of such genes. In fact, HOX proteins have been shown to regulate the development and differentiation of organs both by regulating genes that directly function in morphogenesis and by activating other transcription factors that regulate gene networks involved in morphogenesis. Most of these studies have been performed using Drosophila as a model system (reviewed in [14,15]). However, some progress has been made in defining the gene networks controlled by mammalian HOX proteins during normal cell differentiation. For instance, genes regulated by mouse HOXA13 and HOXD13 during limb formation have been revealed by transcriptome changes in limb tissue at different developmental timepoints [16].

## 2. HOX Genes and Cancer

The correct embryonic development of flies and vertebrates is, in part, mediated by the unique and highly regulated mRNA expression patterns of the HOX genes (Figure 1). All of the genes in each cluster are transcribed in the same direction, which is the opposite of the numbering system. In other words, the direction of transcription of each gene goes away from the high numbered genes and toward the low numbered genes. Therefore, it is the convention in the field to refer to the end of each HOX locus that is nearest the highest number family member (HOXA13) as the 5’ end of the locus and to refer to the end of each HOX locus that is nearest the lowest family member (e.g., HOXA1) as the 3’ end of the locus. The 39 HOX genes are regulated by spatial collinearity, which means that the expression of HOX genes changes along the anterior to posterior axis of the human body; the 3’ HOX genes are highly expressed in the anterior organs whereas the 5’ HOX genes are highly expressed in posterior organs. For example, HOXB13 is required for normal prostate development [17]. Also, loss-of-function mutations of HOXA13 and HOXD13 lead to developmental anomalies of the hands, feet and, in the case of HOXA13, also genitals [18]. The HOX genes are also regulated by temporal collinearity, which means that within each locus the time at which they turn on during development proceeds from the 3’ HOX genes to the 5’ HOX genes. For example, in human pulmonary embryogenesis, the earliest structures, including mainstem bronchi, largely express 3’ HOXA and HOXB family members with progressively more 5’ HOX expression occurring in structures that develop later, such as alveoli [19]. Thus, genes at the 3’ end of clusters are expressed more anteriorly and earlier while genes at the 5′ end of clusters are expressed more posteriorly and later in development. Most of the studies related to the expression patterns of the HOX family have been performed using fruit flies. Of course, the regulation of the HOX genes is more complicated in mammals than in flies because there are four human HOX clusters (as opposed to a single cluster in flies); strict adherence to the spatial and temporal collinearity patterns may not apply in mammalian development. 

**Figure 3:**
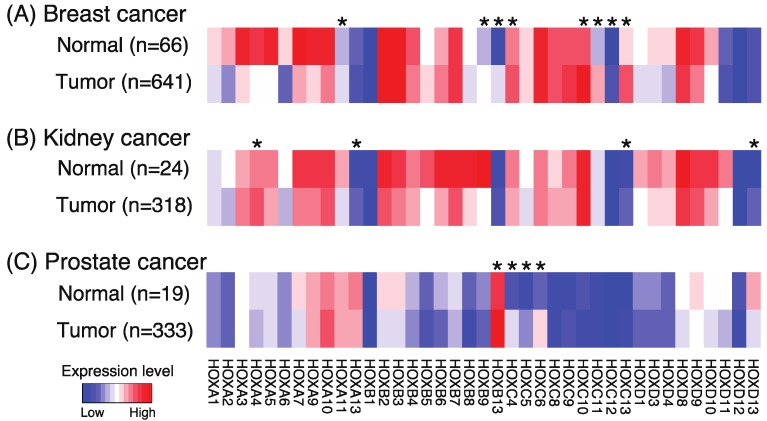
Expression of the 39 human HOX genes in normal vs tumor samples. Shown are heatmaps indicating relative expression levels of the human HOX genes in normal and tumor samples from breast (**A**), kidney (**B**), and prostate (**C**) TCGA RNA-seq datasets. RNA-seq data were normalized as previously described [23]. Wilcoxon rank sum tests were performed between normal and tumor groups, and *p* values were adjusted using the Holm method. An asterisk indicates genes showing a significant upregulation in the tumor samples as compared to the normal samples (adjusted *p* value < 0.01).

Disruptions in the fine-tuned mechanisms regulating HOX gene expression can not only lead to developmental problems but can also lead to human diseases [20]. Specifically, numerous studies have shown aberrant HOX expression in cancer cells. For example, increased expression of HOXA9 is found in most aggressive acute leukemias and is predictive of poor prognosis [21]. In fact, according to an extensive review of the literature [22], only two (HOXC10 and HOXC12) of the 39 human HOX genes had not been reported (at the time of the publication) to be aberrantly expressed in a solid tumor across 10 tissue types; the two HOX genes that were reported to be most commonly altered in solid tumors are HOXA9 and HOXB13. A comparison of the levels of the 39 HOX family members in a large number of normal vs. tumor samples from The Cancer Genome Atlas (TCGA) reveals that different HOX genes are upregulated in different cancer types (Figure 3). Notably, both HOXC10 and HOXC12 show increased expression in the breast tumor samples; thus, all HOX genes show altered expression in some form of cancer.

Studies have shown that family members in the HOXB and HOXC loci are strongly associated with prostate cancer. For example, missense germline HOXB13 mutations have been associated with early-onset prostate cancer, breast cancer, and colorectal cancer [24,25,26]. HOXB13 expression has been linked to robust cell growth and migration in response to androgens in prostate cancer cells [27] and HOXB13 has been identified as an androgen receptor-interacting protein [28,29]. Chen et al. [30] also showed that overexpression of HOXB13 predicts poor patient survival and promotes proliferation and migration of prostate cancer cells. An analysis of the 39-member panel of HOX genes [31,32,33] revealed that HOXC4, HOXC5, HOXC6, and HOXC8 are overexpressed in prostate cancer cell lines and in the small set of examined prostate tumors (see also Figure 3C for an analysis of a larger set of prostate tumors). An unbiased analysis of DNA methylation and gene expression data using a large set of tumors from TCGA identified HOXB13, HOXC4, HOXC5, and HOXC6 in the set of the top 10 transcription factors whose expression is linked to the creation of newly active distal regulatory elements in prostate cancers [23]. The members of the HOXC locus are of particular interest relative to prostate cancer because not only have investigators shown that reduction in levels of HOXC proteins reduce proliferation of prostate cancer cell lines [34], but HOXC family members have also been shown to be clinically relevant biomarkers. For example, Leyten et al. [35] developed an 8-gene panel and a 3-gene panel that can identify patients with aggressive prostate cancer; notably HOXC6 and HOXC4 were in the 8-gene panel whereas HOXC6 remained as an important classifier in the smaller 3-gene panel. Others have developed a 16-gene panel [36] and a 5-gene panel [37] of prostate cancer markers that include HOXC6; both panels can predict recurrence of prostate cancer after treatment. Altered expression of HOX family members has been associated with prognostic diagnoses for other cancers. According to The Human Protein Atlas [38], poor prognosis of patients with renal cancer is associated with higher levels of HOXA3, HOXA11, HOXC6, HOXC8, and HOXD10 expression, whereas favorable prognosis of patients with renal cancer is associated with increased levels of HOXA6, HOXA7, and HOXB8 expression. Also, poor prognosis of patients with endometrial cancer is associated with higher levels of HOXA4, HOX5, HOXA6, HOXA7, and HOXB9 expression, whereas favorable prognosis of patients with endometrial cancer is associated with higher levels of HOXB5 and HOXB6 expression. Increased expression of HOX proteins has also been associated with poor prognosis of patients with glioma and lung, liver, colorectal, head and neck, and ovarian cancers.

Clearly, aberrant expression of certain HOX proteins can serve as biomarkers and/or drivers of neoplastic transformation. However, it is not yet understood if there is specificity of action of the different HOX paralogs or HOX loci or if upregulation of different HOX family member would have similar consequences in a particular tissue (e.g., would HOXA6 cause a similar neoplastic transformation of normal prostate cells as does HOXC6 if it was artificially expressed at high levels?). If so, this might suggest that the observed relationship of a particular HOX family member to a certain type of cancer may be a consequence of a tumor-specific alteration in the overall transcriptional activity of a particular HOX locus rather than due to functional differences in the HOX members from the different loci. Specifically, it is not clear if HOXC4, HOXC5, and HOXC6 are all drivers of prostate cancer or if the three proteins are all upregulated due to genomic proximity, but only one is a direct regulator of tumorigenesis. It is also not yet understood how an upregulated HOX family member affects the normal functioning of other HOX family members. For example, do inappropriately high levels of one HOX family member in tumors result in competition at regulatory elements with the HOX family member that normally drives prostate tissue differentiation? If so, this could lead to a downregulation of differentiation-specific target genes via a dominant negative mechanism. Alternatively, does the upregulated HOX bind to and activate a completely different set of genes whose function interferes with differentiation? Rather than competition (and down-regulation of normal HOX target genes), this second mechanism would lead to inappropriate upregulation of other genes. Such issues are also clouded by the fact that ectopic expression of certain Xenopus Hox proteins have been shown to lead to increased expression of other Hox genes transcribed from the same or different cluster [39]. It is critical that questions addressing how HOX proteins influence the activity and/or regulation of other HOX family members be investigated to gain a deeper understanding of how a HOX gene that is normally involved in very specific morphological differentiation can transform into a transcription factor that promotes the de-differentiated phenotype of cancer cells.

## 3. Future Directions and Technological Challenges

### 3.1. Solving the HOX Specificity Paradox

The term homeobox is used to describe this gene family because aberrant expression of these genes can, in certain situations, result in homeosis, i.e., the replacement of one organ with another [40]. However, it should also be noted that the subgroup of homeobox genes encoded at the 4 HOX loci are derived from two rounds of genomic duplication and thus the 39 HOX genes share highly similar protein sequences (Figure 2 and Figure S1). This high degree of protein similarity leads to the “HOX specificity paradox”: how can such structurally similar proteins have distinct functions? Perhaps the paralogs from the four HOX loci (e.g., HOXA13, HOXB13, HOXC13, and HOXD13) have a similar function but specificity comes when comparing the different numbered genes (e.g., HOXA6 vs HOXA13). Unfortunately, to date, very few studies have compared the function of different HOX genes in mouse or human cells. We note that the International Mouse Phenotype Consortium [41] is creating mice having knockout alleles for Hox proteins. Currently, the Consortium lists mice lacking one allele of Hoxa2, Hoxa10, Hoxb13, Hoxc11, or Hoxc12. It would be interesting to knockout one HOX gene in a normal, differentiated cell and/or in an abnormal, cancer cell and then upregulate other HOX genes to see if they could compensate for the loss. As described above, there is evidence that some mouse HOX paralogs can substitute for each other in kidney development. However, functional equivalence is not always the rule. For example, HOXA5 has an inhibitory effect on endothelial cell differentiation whereas HOXB5 promotes differentiation [6,42,43].

Because the DNA binding homeodomain is highly similar amongst the HOX proteins (especially when comparing paralogs), it has been proposed that protein-protein interactions mediated by amino acids outside of the DNA binding domain may confer functional specificity. For example, Pre-B-cell Leukemia Homeobox (PBX) and Myeloid Ecotropic Viral Integration Site 1 Homolog (MEIS) proteins, which are members of another homeobox protein subgroup referred to as Three Amino Acid Extension (TALE) proteins, have been shown to interact with a hexapeptide motif in the N terminus of HOX proteins [4]; MEIS family members bind to HOX paralogs 9-13 and PBX family members bind to HOX paralogs 1–11 [44,45,46,47]. Aligning the sequences adjacent to the hexapeptide has identified amino acids that are conserved within paralog groups and it has been suggested that these paralog-specific amino acids may fine tune HOX-PBX interactions and provide DNA binding specificity [48]. Similar, but distinct, partners may confer additional specificity of binding via a functional cooperation with various HOX proteins (reviewed in [6]). Further investigation of functional specificity of the 39 HOX genes requires a thorough analysis of their protein interaction partners. For example, HOX-interacting proteins could be identified by immunoprecipitation followed by mass spectrometry using tagged constructs or antibodies to endogenous proteins; a tagged HOXC9 has been used to identify E2F6 as an interacting protein [49] and an HA-tagged HOXA9 has been used to identify MEIS1, PBX2, PBX3, C/EBP, and STAT5 as interacting proteins [50,51]. Others have used the proximity ligation assay to identify proteins in close proximity to HOX family members in cultured cells; specifically, HOXA10 has been shown to colocalize with CTCF [52]. Additionally, bimolecular fluorescence complementation (BiFC), which measures a global interaction of two transcription factors in the context of genomic DNA, has characterized interactions properties between HOX, PBX1, and MEIS homeodomain proteins [53].

#### Technological Challenges

A major problem with identifying interaction partners of the HOX proteins is that the HOX proteins are generally expressed at fairly low levels, even in cancer cells. Because of the low expression levels, very large amounts of starting nuclear extract are required for mass spectrometry experiments. Another problem is that most commercial antibodies to the HOX proteins are of very low quality (perhaps due to the strong evolutionary conservation of their protein sequences) and cannot even detect exogenously expressed high levels of the target HOX protein. Due to these two issues, it is likely that identification of interacting partners will require the use of overexpressed, tagged proteins. Such assays come with the caveats that overexpression may alter the normal stoichiometry of components in a complex and the tag may alter protein folding and/or physically interfere with certain protein-protein interactions. However, comparisons of different tags placed either at the N- or C-terminus, introduction of the tag into the endogenous locus, or carefully controlled expression of the tagged protein may partially alleviate these problems. Another issue could be that identification of bona fide HOX-interacting proteins may require that HOX proteins be bound to their genomic target sites. For example, a structural change may occur upon binding of a HOX protein to DNA that uncovers a protein interaction domain and/or a second DNA binding protein may be required to cooperate with the HOX protein to co-recruit a third protein. To address this issue, one can use Rapid Immunoprecipitation Mass spectrometry of Endogenous proteins (RIME) [54]. This method identifies interaction partners of chromatin-bound proteins rather than using soluble nuclear extracts for the immunoprecipitation step. Although this method has not yet been attempted with a HOX protein as the antibody target, RIME has been used to identify HOXB13 as an interaction partner of the androgen receptor [28].

### 3.2. Identifying Target Genes of the Human HOX Proteins

Because of their importance in normal development and their link to diseases such as aggressive prostate cancer, identifying regulatory elements bound by the HOX proteins should be of high priority to the scientific community. However, of the 39 human HOX proteins, only a few have been studied using genome-wide technologies. Because HOXB13 is upregulated in prostate cancer, these cells have been used to identify HOXB13 binding sites using ChIP-seq [28,29,55]. Binding sites for HOXC9 (in neuroblastoma cells) and HOXC6 (in prostate cancer cells) have been identified using an antibody to a tagged, exogenously expressed protein [49,56]. Modest success has been reported for the identification of binding sites for mouse HOX proteins. To date, endogenous antibodies have been used to identify binding sites for mouse HOXA13, HOXD13, HOXA2, and HOXB4 [16,57,58] and tagged constructs have been used to identify binding sites for mouse HOXA9 and HOXC9 [21,50,59,60]. Taken together, these initial studies have shown that although some of the binding sites are in promoter regions, the majority of HOX binding sites are located in distal regulatory elements within intergenic and intronic regions (>20 Kb from a transcription start site). The distal binding sites have a high overlap with the active enhancer mark H3K27Ac. As is consistent with the activity of a transcription factor that binds to enhancers, it has been suggested that HOX proteins could possibly regulate multiple steps of transcription (reviewed in [61]). For example, the HOX proteins could function as pioneer transcription factors that can assist in switching histone marks from inactive to active by recruiting chromatin remodelers, such as Trithorax group and Polycomb group proteins, to distal regulatory elements. Also, HOX proteins may influence enhancer-promoter looping by affecting CTCF and GAGA (a Trithorax group protein) binding. Additionally, because some of the binding sites are in proximal promoter regions, HOX proteins could possibly interact with the RNA Polymerase II initiation complex and/or affect mRNA processing.

#### Technological Challenges

Clearly, a genomic binding profile analysis of all 39 human HOX proteins in a variety of different normal and diseases tissues (such as cancer) would be highly instructive. Motifs identified using in vitro and in vivo methods [62,63,64] indicate that HOX binding sites are short AT-rich sequences (e.g., TAAT or TTAT). Sequences similar to this motif are found throughout the genome and it is likely that additional information is required to specify in vivo binding of a HOX family member. As discussed above, cooperative binding with other proteins may help to direct a HOX protein to a subset of the AT-rich HOX motifs found throughout the genome. If so, then a search for HOX interaction partners is critical. In addition to the mass spectrometry methods described above, another method by which cooperative binding partners can be identified is to perform motif analysis of in vivo binding sites and look for motifs for other transcription factors. For example, a GATA3 motif was identified through analysis of TCF7L2 ChIP-seq peaks from breast cancer cells [65]; follow-up studies demonstrated that GATA3 and TCF7L2 interact with each other and that depletion of GATA3 in breast cancer cells reduces binding of TCF7L2 to specific sites. Similar studies may reveal tissue-specific interaction partners for HOX proteins. Many HOX-bound enhancers have multiple low affinity binding sites that are called homotypic binding clusters. It has been suggested that specificity of HOX protein recruitment could be achieved by using a cluster of low affinity HOX binding sites even if HOX binding to individual motifs is less precise [66]. Support for this mechanism could be obtained from a motif analysis of the genomic neighborhood of a set of in vivo HOX binding sites; clusters of AT-rich motifs in the neighborhood of the peak centers may be present at the robust peaks. However, what is clearly needed to understand HOX in vivo binding specificity is many more ChIP-seq datasets. The lack of published ChIP-seq data for most human HOX proteins (and the absence of HOX ChIP-seq data in databases from genomic consortia such as ENCODE) could possibly be due to the low expression level of the proteins and lack of high-quality commercial antibodies. However, if so, this could be alleviated by transiently expressing higher levels of tagged factors. We suggest that the creation of a library of tagged, inducible constructs for all 39 HOX proteins would be a useful addition to the field. It is also possible that the paucity of published HOX ChIP-seq data has to do with a short residence time of the HOX proteins on the chromatin. If so, then technological modifications to the standard ChIP assay may be needed. For example, Sheth et al. [16] used the protein-protein crosslinker DSG, in combination with formaldehyde, to identify genomic binding sites for mouse HOXA13 and HOXD13. Alternatively, methods, such as DAM-ID [67], which allow the identification of binding sites through the deposition of a mark (adenine DNA methylation in the case of DAM-ID) over time may solve the problem. Lacin et al. [68] have used DAM-ID (in combination with microarrays) to identify binding sites of the Drosophila homeodomain protein Hb9 but this approach has not yet been attempted for human HOX proteins. Newer genome-wide immunoprecipitation-based methods, such as DAM-IP [69], may be useful for identifying genomic binding sites of the human HOX proteins. A DAM-IP HOX construct could be created at the endogenous locus in a given cell type using CRISPR-mediated gene editing. However, this would limit the binding site identification to that cell line. Similar to the tagged expression constructs described above, a library of inducible HOX proteins fused to an enzyme that can mark the environment of a HOX genomic binding site would be useful for the field.

### 3.3. Developing Inhibitors that Can Restore Normal Cellular Function in Cells Harboring Aberrantly Expressed HOX Family Members

As noted above, numerous HOX proteins have been shown to be upregulated in tumors and can serve as robust biomarkers for clinical diagnosis and treatment [21,22,23,33,35]. Importantly, evidence also suggests that certain HOX genes are drivers of tumorigenesis. For example, several studies have shown that reduction of levels of an overexpressed HOX can move cancer cells towards a more normal phenotype. Specifically, depletion of HOXB13 decreased proliferation of cancer cells [30], siRNA-mediated reduction of HOXC6 reduced proliferation of prostate cancer cells [34], siRNA knockdown of HOXB13 reduced cell growth and migration of prostate cells in response to androgen treatment [27], and siRNA knockdown of HOXD9 reduced glioma cancer proliferation and colony formation but increased apoptosis [70]. These studies demonstrate that, at least in some cases, HOX genes can be drivers of tumorigenesis and are not simply upregulated as a consequence of neoplastic transformation. Such studies suggest that inhibition of HOX levels or activity may be a rationale therapeutic option.

#### Technological challenges

Although it may seem logical to attempt to develop direct inhibitors of HOX proteins that could reduce their activity in cancer cells, transcription factors are thought to be quite difficult to target in this way. Encouragingly, Morgan and colleagues have developed a cell permeable 18-amino acid peptide (called HXR9) that can disrupt (and thus functionally inactivate) interaction of a subset of HOX proteins (members of paralogue groups 1-9) with a common cofactor (PBX). They show that HXR9 can block the growth of prostate tumors in a mouse xenograph model system [32]. HXR9 has also been shown to inhibit the growth of a range of other tumor types in mouse xenograft models (see Table 1 in [71]). To date, large-scale screening experiments for small molecule inhibitors of human HOX proteins have not yet been reported. However, a recent study has reported using a chemical library consisting of 500,000 small molecules to identify a compound (CSRM617) that binds to the homeodomain protein ONECUT2 and prevents binding of ONECUT2 to DNA [72]. The investigators further showed that CSRM617 can inhibit cell growth and induce apoptosis in vitro in several prostate cancer cell lines that express moderate to high levels of ONECUT2 and that the compound can suppress prostate cancer growth and metastasis in a nude mouse model system. Thus, it would perhaps be useful to screen chemical libraries for inhibitors of the HOX proteins. Methods other than directly inhibiting the function of a HOX protein could also be explored. For example, the level of a HOX protein could be reduced using siRNA to target HOX transcript levels, genomic nucleases based on zinc fingers or the CRISPR/Cas9 system could be used to delete all or part of a HOX coding sequence, or epigenetic toggle switches could be used to reduce the activity of the promoter region of a HOX gene by deposition of repressive histone marks. Although some of these methods have entered into clinical treatment for other genes, they are not considered as robust as using a small molecule inhibitor. Another approach could be to inhibit the activity of an enzyme that is required for HOX expression. Inhibition of MYC function has been achieved by targeting a component of a co-activator complex that regulates the MYC oncogene. MYC expression has been shown to be driven by BET bromodomain proteins, which bind to acetylated histone tails and facilitate transcriptional activation. JQ1, a small molecule inhibitor of the BET bromodomain, can downregulate MYC expression and, as a consequence, downregulate MYC target genes [73]. We do not yet know all of the regulatory elements and proteins that control HOX gene expression; 3-dimensional chromatin interaction data and epigenomic mapping in cancer cells may provide useful information. Finally, the identification of gene networks that are activated by an aberrantly expressed HOX protein may identify more easily druggable enzymes whose activity is responsible for the HOX-mediated disease phenotype. For example, Morsi El-Kadi et al. have shown that Xenopus Hoxb4 is a negative regulator of XRap1, which itself antagonizes Ras signaling [74]. This suggests that increased expression of Hoxb4 may lead to upregulation of Ras; in this example, targeting the Ras pathway may be one approach to inhibit Hoxb4.

## 4. Conclusions

The 39-member human HOX family is important for normal development and has been implicated in the initiation and progression of human diseases. However, this family is severely under-studied, likely due to idiosyncratic details of their structure, expression, and function. We suggest that a concerted and collaborative effort to produce genome-wide binding profiles, identify interacting partners, and develop HOX network inhibitors would lead to a deeper understanding of human development and disease (Box 1).

Box 1 **The next phase of HOX exploration.**

**Q1. Do HOX proteins have post-DNA binding specificity or is ostensible specificity achieved mainly via temporal and spatial expression patterns?**

**What is needed to answer this question:**

Comprehensive genome-wide mapping of the 39 human HOX proteins in multiple tissues and disease states

Comprehensive identification of protein partners of HOX family members in the nucleoplasm and when bound to chromatin

**Q2. Do HOX proteins represent a relatively untapped cohort of potential therapeutic targets?**

**What is needed to answer this question:**

Agents that inhibit the function of specific HOX family members or the activity of components of specific HOX-mediated networks.

Expanded analysis of HOX proteins as disease-specific biomarkers and initiation of clinical trials of existing (and future) HOX-related inhibitors.

## Supplementary Materials

The following are available online at https://www.mdpi.com/2072-6694/11/3/323/s1, Figure S1: Phylogenetic analysis of the 39 human HOX genes, Table S1. Distance values comparing all HOX proteins and all HOX protein homeodomains.
