# Supplementary material for: The Enigmatic HOX Genes: Can We Crack Their Code?
Source: Cancers (Basel). 2019 Mar 7;11(3):323. doi: 10.3390/cancers11030323 (PMC6468460; doi:10.3390/cancers11030323)
Supplement: Supplementary file 1 [file cancers-11-00323-s001.zip › FigureS1_and_legend.pdf]

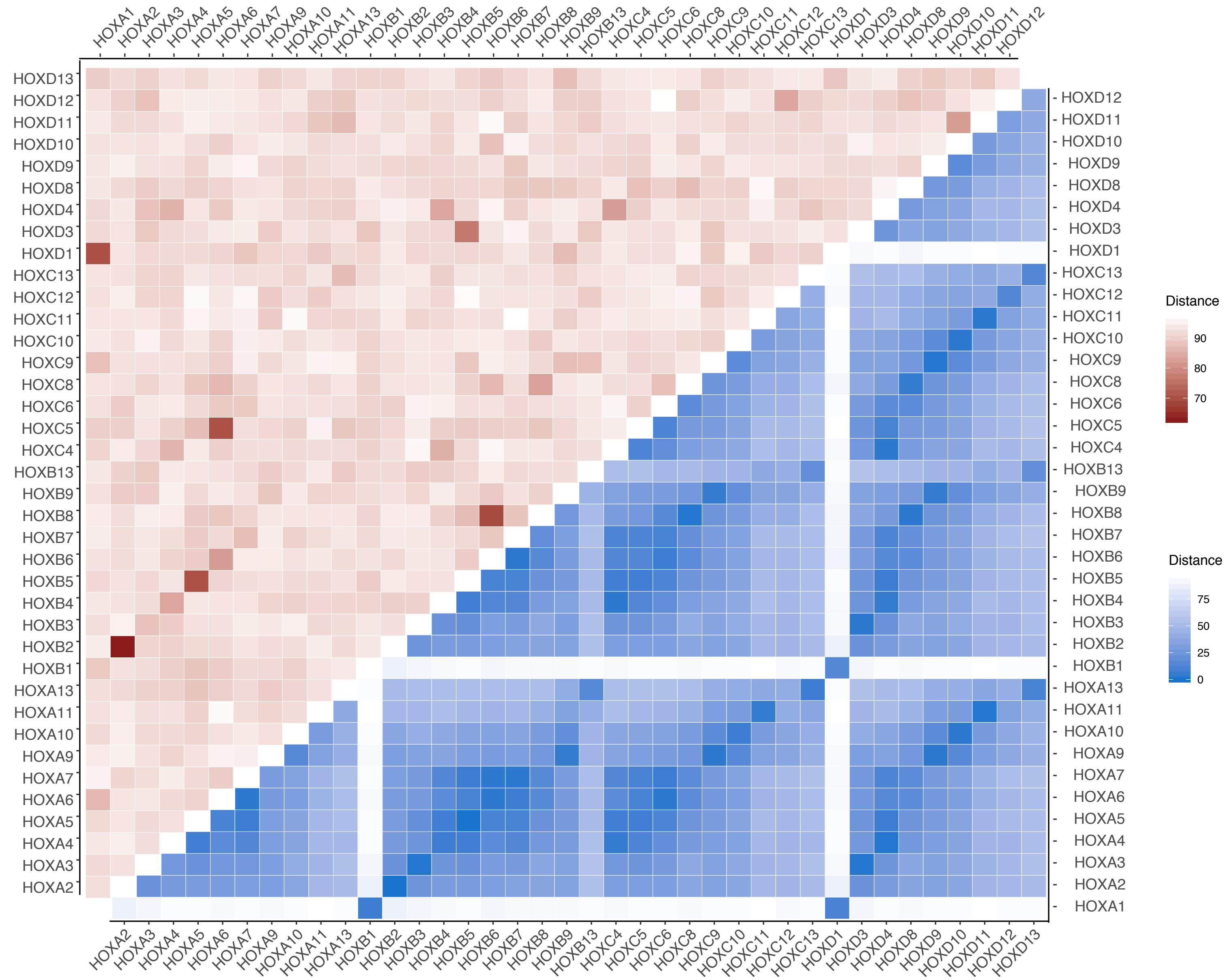

Luo: Supplemental Figure S1. Phylogenetic analysis of the 39 human HOX genes. Shown is distance matrix comparing the full-length protein sequences of all HOX proteins (left top triangle; pink squares) and a distance matrix comparing the homeodomain sequences of all HOX proteins (right bottom triangle; blue squares); matrices were created using distmat (www.bioinformatics.nl/cgi-bin/emboss/distmat/). Color legend: Distance: the darker the color, the more similar are the two proteins and the lighter the color the less similar are the two proteins. The HOX sequences were retrieved from RefSeq (ncbi.nlm.nih.gov/refseq/) and the homeodomains were annotated using Pfam (pfam.xfam.org). See Table S1 for all distance values.
